# Quantitative proteomic analysis of *Shigella flexneri* and *Shigella sonnei* Generalized Modules for Membrane Antigens (GMMA) reveals highly pure preparations

**DOI:** 10.1016/j.ijmm.2015.12.003

**Published:** 2016-02

**Authors:** Luana Maggiore, Lu Yu, Ulrich Omasits, Omar Rossi, Gordon Dougan, Nicholas R. Thomson, Allan Saul, Jyoti S. Choudhary, Christiane Gerke

**Affiliations:** a Novartis Vaccines Institute for Global Health, Via Fiorentina 1, 53100 Siena, Italy; b Wellcome Trust Sanger Institute, Genome Campus, Hinxton, Cambridge CB10 1SA, UK

**Keywords:** −p, absence of virulence plasmid (either pSS or pINV), +p, presence of virulence plasmid (either pSS or pINV), Cyto, cytoplasm, CytoM, cytoplasmic membrane, DOMV, detergent-derived outer membrane vesicles, *E. coli*, *Escherichia coli*, GMMA, Generalized Modules for Membrane Antigens, NOMV, native outer membrane vesicles, OM, outer membrane, OM lipo, outer membrane lipoprotein, OMV, outer membrane vesicles, OAg, O antigen, PP, periplasm, SD, standard deviation, Sf2a, hyperblebbing plasmid-cured OAg-deficient *Shigella flexneri* 2a 2457T, SHIFL, *Shigella flexneri* protein database, mixed serotypes, SHIF8, *Shigella flexneri* serotype 5b protein database, SHISO, *Shigella sonnei* 53G protein database, SHISS, *Shigella sonnei* 046 protein database, SOMV, spontaneous outer membrane vesicles, Ss, hyperblebbing plasmid-cured OAg-deficient *Shigella sonnei* 53G, vs, *versus*, *Shigella sonnei*, *Shigella flexneri* 2a, Proteomics, GMMA, Vaccine, iBAQ

## Abstract

Outer membrane blebs are naturally shed by Gram-negative bacteria and are candidates of interest for vaccines development. Genetic modification of bacteria to induce hyperblebbing greatly increases the yield of blebs, called Generalized Modules for Membrane Antigens (GMMA). The composition of the GMMA from hyperblebbing mutants of *Shigella flexneri* 2a and *Shigella sonnei* were quantitatively analyzed using high-sensitivity mass spectrometry with the label-free iBAQ procedure and compared to the composition of the solubilized cells of the GMMA-producing strains. There were 2306 proteins identified, 659 in GMMA and 2239 in bacteria, of which 290 (GMMA) and 1696 (bacteria) were common to both *S. flexneri* 2a and *S. sonnei*. Predicted outer membrane and periplasmic proteins constituted 95.7% and 98.7% of the protein mass of *S. flexneri* 2a and *S. sonnei* GMMA, respectively. Among the remaining proteins, small quantities of ribosomal proteins collectively accounted for more than half of the predicted cytoplasmic protein impurities in the GMMA. In GMMA, the outer membrane and periplasmic proteins were enriched 13.3-fold (*S. flexneri* 2a) and 8.3-fold (*S. sonnei*) compared to their abundance in the parent bacteria. Both periplasmic and outer membrane proteins were enriched similarly, suggesting that GMMA have a similar surface to volume ratio as the surface to periplasmic volume ratio in these mutant bacteria. Results in *S. flexneri* 2a and *S. sonnei* showed high reproducibility indicating a robust GMMA-producing process and the low contamination by cytoplasmic proteins support the use of GMMA for vaccines. Data are available via ProteomeXchange with identifier PXD002517.

## Graphical abstract

**Figure:**
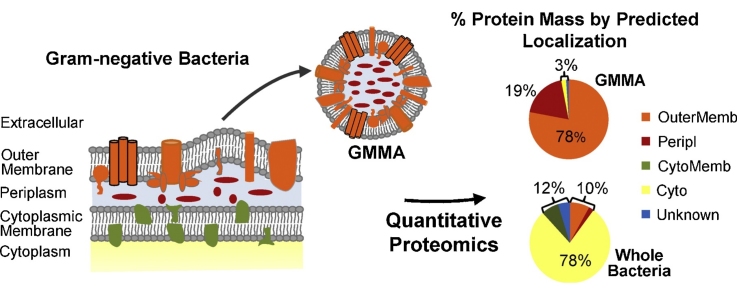

## 1 Introduction

*Shigellae* are Gram-negative enterobacteria classified into fifty different serotypes based on the carbohydrate composition of the outer polysaccharide antigen (O antigen, OAg) of the lipopolysaccharide (LPS) (Levine et al., 2007). The worldwide dominant serotypes are *S. sonnei* and *S. flexneri* 2a (Levine et al., 2007, Chang et al., 2012, Livio et al., 2014). They are the etiological agent of shigellosis, one of the most frequent causes of medium and severe diarrhea in developing countries, especially in children under five years of age (Kotloff et al., 2013). Currently, no *Shigella* vaccine is widely available.

Gram-negative bacteria naturally shed outer membranes in a process of blebbing. These particles are known as native outer membrane vesicles (NOMV), spontaneous outer membrane vesicles (SOMV), or sometimes just OMV. They are quite different from the outer membrane vesicles derived by detergent extraction of whole bacteria (also usually called OMV or sometimes DOMV) and used in meningitis vaccines (Holst et al., 2013), due to the depletion of lipoproteins and lipooligosaccharides by the detergent from DOMV (Ferrari et al., 2006, van de Waterbeemd et al., 2013). The composition of NOMV reflects the composition of the outer membrane of the donor bacteria, an important interface between the bacterial cell and its environment, and thus outer membrane blebs represent ideal candidates for vaccine development (Ellis and Kuehn, 2010). Blebs present these antigens in the natural membrane context and in the presence of powerful stimulators of the innate immune system (Ferrari et al., 2006, Park et al., 2011, Ellis et al., 2010) and elicit protection in animal models against bacterial infections (Alaniz et al., 2007, Schild et al., 2008, Park et al., 2011), including *Shigella* (Camacho et al., 2011, Mitra et al., 2013). Therefore NOMV have been proposed for use as vaccines (Ellis and Kuehn, 2010). However, NOMV are generally present at low concentrations in bacterial cultures. Using genetic modification, it is possible to induce high level shedding of blebs (“gemma” in Italian), called Generalized Modules for Membrane Antigens (GMMA) (Berlanda Scorza et al., 2012), that together with high-yield, industrial processes form the bases for a practical vaccine platform (Berlanda Scorza et al., 2012, Gerke et al., 2015).

Several studies have identified proteins in the proteome of outer membrane blebs, e.g. from *Escherichia coli* (Berlanda Scorza et al., 2008), *Helicobacter pylori* (Mullaney et al., 2009), *Edwardsiella tarda* (Park et al., 2011), *Francisella novicida* (Pierson et al., 2011), *Neisseria meningitidis* (van de Waterbeemd et al., 2013) and *Haemophilus influenza* (Roier et al., 2015). These studies, including our previous analysis of *Shigella sonnei* GMMA (Berlanda Scorza et al., 2012), conducted qualitative analyses of the proteome to document the proteins detectable. When the results were expressed by number of proteins identified, cytoplasmic proteins that are not predicted to be included in outer membrane blebs comprised a substantial proportion of the total (Berlanda Scorza et al., 2008, Berlanda Scorza et al., 2012, Choi et al., 2011). The actual proportion appears to depend on the sensitivity of the method (2D gel-based proteomics (Berlanda Scorza et al., 2008, Berlanda Scorza et al., 2012) versus LC–MS/MS (Choi et al., 2011)) and the sensitivity of the detection. The higher the sensitivity, the more low abundant cytoplasmic proteins are detected and thus the smaller the detected proportion of predicted periplasmic and outer membrane proteins.

Two studies used labeling techniques to quantitatively compare the ratio of the quantity of each of the detectible proteins in two or more related membrane samples from *N. meningitidis*. Chemical isotopic labeling using heavy (CD_2_O) or light (CH_2_O) formaldehyde has been used to measure the ratio of individual proteins in spontaneously released (SOMV), chelate induced (NOMV) and detergent extracted (DOMV) vesicles from an unencapsulated recombinant B group *Neisseria meningitis* carrying deletions of *galE* to truncate the LOS, *lplX* to give a penta-acyl Lipid A structure and *rmpM* to increase spontaneous OMV production (van de Waterbeemd et al., 2013). Metabolic labeling with ^15^N was used in another study of unencapsulated *N. meningitidis* to quantitatively measure the relative abundance of proteins in SOMV with outer membrane preparations (Lappann et al., 2013). Neither of these methods provides quantitative information on the abundance of each protein present within a single sample. For developing outer membrane vaccines, we need to know the relative mass of each protein present in a single vaccine preparation, especially for the outer membrane and periplasmic proteins.

One study, the proteomic analysis of detergent extracted and spontaneously released OMV of *H. influenza* (Roier et al., 2015), used a label free technique to rank proteins within each sample on the basis of their Mascot ion scores. While this a measure of the probability that the protein derived peptides are correctly identified it is not a direct measure of the relative abundance.

Two studies have used label free quantification of proteins present in OMV to estimate the relative amount of all the proteins detected:

• The Lappman et al. study (Lappann et al., 2013) quoted above, in addition to using metabolic labeling to determine ratio in different preparations, also used a non-labeled technique, normalized spectral abundance factors (Zybailov et al., 2006) to estimate the relative amounts of each protein within each sample.
• The abundance of proteins in spontaneously released OMV from *Pseudomonas aeruginosa* (Choi et al., 2011), was measured using the APEX tool (Lu et al., 2007).

As part of a program of developing a GMMA-based *Shigella* vaccine (Gerke et al., 2015), we used the iBAQ index (Schwanhäusser et al., 2011) for the non-labeled, quantitative proteomic analysis of GMMA from hyperblebbing *S. sonnei* 53G (Ss) and *Shigella flexneri* 2a 2457T (Sf2a) strains and show that both preparations contain low levels of cytoplasmic proteins, supporting the use of GMMA as vaccines for delivering outer membrane and periplasmic antigens.

## 2 Materials and methods

### 2.1 *Shigella* strains and bacterial growth condition

In this study we used previously described hyperblebbing (Δ*tolR*) O antigen-deficient strains of *S. sonnei* 53G and *S. flexneri* 2a 2457T that were cured of the virulence plasmid: *S. sonnei* −p Δ*tolR* (Berlanda Scorza et al., 2012) (in this study abbreviated as Ss) and *S. flexneri* 2a −p Δ*tolR* Δ*rfbG* (Rossi et al., 2014) (in this study abbreviated as Sf2a).

The defined medium for *S. sonnei* was described previously (Berlanda Scorza et al., 2012) and had the following composition: 30 g/L glycerol, 2.5 g/L aspartic acid, 13.3 g/L KH_2_PO_4_, 1.2 g/L (NH_4_)_2_HPO_4_ 4 g/L, MgSO_4_*7H_2_O, 1.7 g/L citric acid, 2.5 mg/L CoCL_2_*6H_2_O, 15 mg/L MnCl_2_*4H_2_O, 1.5 mg/L CuCl_2_*2H_2_O, 3 mg/L H_3_BO_3_, 2.5 mg/L Na_2_MoO_4_*2H_2_O, 13 mg/L Zn(CH_3_COO)_2_*2H_2_O, 2 μM ferric citrate, 50 mg/L thiamine, 10 mg/L nicotinic acid. For Sf2a, 4 g/L of glucose was also added. Both strains were grown at 37 °C.

### 2.2 Preparation of GMMA and solubilized bacteria

To produce GMMA, the strains were grown in medium supplemented with antibiotics (30 μg/mL kanamycin for Ss; 30 μg/mL kanamycin and 100 μg/mL erythromycin for Sf2a) to OD_600nm_ = 0.3. The cultures were then diluted into 1 L of fresh medium (without antibiotics) to a starting OD_600nm_ of 0.05 and incubated at 37 °C. After growing to OD_600nm_ of 0.4, in case of Ss, and of 0.3, in case of Sf2a, cultures were centrifuged for 15 min at 5000 × *g* and the supernatants removed for GMMA preparation.

The bacterial pellets were solubilized after two washes with cold phosphate buffered saline (PBS) by resuspending in lysis buffer (0.8 M urea, 0.4% SDS, 1 mM DTT and 20 mM Tris, pH 7.4) to prepare lysates.

The culture supernatants were filtered through a 0.22 μm filter unit (Millipore). The filtered samples were concentrated in a Stirred Cell Model 8400 (Millipore) through a 100,000 Da regenerated cellulose membrane (Millipore). GMMA were then separated from soluble proteins by ultracentrifugation at 186,000 × *g* for 2 h at 4 °C (Optima™ L-series, 45Ti rotor, Beckman Instruments). The GMMA pellets were washed once with cold PBS, resuspended in 20 mM Tris, pH 7.4, then filtered through a 0.22 μm filter.

### 2.3 Total protein quantification

The protein concentration in GMMA and solubilized bacteria was determined using Pierce Microplate BCA Protein Assay Kit-Reducing Agent Compatible (Thermo Scientific). Prior to the determination GMMA were diluted into the buffer used to prepare the solubilized bacteria.

### 2.4 SDS-PAGE

GMMA (13 μg) and cell lysates (26 μg) were mixed with NuPAGE SDS sample buffer (Invitrogen) containing 5 mM Tris(2-carboxyethyl)phosphine hydrochloride solution (TCEP, Sigma), boiled for 10 min, and iodoacetamide (IAA, Sigma) was then added to a final concentration of 10 mM. Following 30 min incubation in the dark at room temperature, the samples were loaded into NuPAGE^R^ 12% Bis-Tris gels using SeeBlue Plus 2 Pre-Stained Standard (Invitrogen) for molecular weight calibration and MOPS as running buffer (Invitrogen). Following electrophoresis, the gel was then fixed with 40% methanol (MeOH), 2% acetic acid for 30 min stained overnight with Brilliant Blue G-colloidal solution (Sigma), and destained with 30% MeOH. Gels were scanned with an Epson Expression 1640XL scanner.

### 2.5 In-gel protein digestion

Each lane of the gel was excised in 15–16 slices; bands and blank regions were excised separately. Each slice was then cut into small pieces and transferred into a 96-well pierced plate (Proxeon) stacked on a normal plate. The gel pieces were destained with 50% 50 mM triethylammonium bicarbonate buffer (TEAB) pH 8.0, 50% acetonitrile for 30 min at 37 °C with shaking. The liquid was removed by centrifugation of the Proxeon plate. The destaining step was repeated until the gel pieces were completely white. The samples were incubated with acetonitrile, the liquid was removed, and the plate was air-dried. The gel pieces were digested with 0.15 μg/well of trypsin. Prior to digestion 50 fmol/well of enolase were added as internal standard for the label-free quantification. After 2 h of digestion at 37 °C, MeOH was added to give a final concentration of 33% and another 0.15 μg/well of trypsin was added to each well and the digestion was performed for additional 3 h at 37 °C, followed by 25 °C overnight. Peptides were extracted at 37 °C using one change of 50% acetonitrile (20 min), 2 changes of 50% acetonitrile/0.25% formic acid (30 min each), and one change of pure acetonitrile (30 min), dried, and stored frozen at −20 °C until analysis.

### 2.6 LC/MS–MS analysis

The samples were analyzed with on-line nano LC–MS/MS on an Ultimate 3000 RSLCnano System (Dionex) coupled to a LTQ Orbitrap Velos (Thermo Fisher) hybrid mass spectrometer equipped with a nanospray source. Peptides were separated on a 75 μm id × 50 cm PepMap RSLC column (2 μm, Dionex) over a 120 min linear gradient of 4–32% CH_3_CN/0.1% FA. The LTQ Orbitrap Velos mass spectrometer was operated in the “top 10” data-dependent acquisition mode. The 10 most abundant multiply-charged precursor ions in the MS survey scan in the Orbitrap (*m*/*z* 400–1500, with the lock mass at 445.120025), with a minimal signal above 2000 counts, were dynamically selected for Collision induced Dissociation (MS/MS) in the LTQ Velos ion trap. The preview mode of FT master scan was disabled. The Orbitrap resolution was set to 60,000 at *m*/*z* 400. The dynamic exclusion was set to a mass width of ±20 ppm and a duration of 45 s. To achieve high mass accuracy, the AGC (Automatic Gain Control) was set to 1 × 10^6^ for the full MS survey in the Orbitrap, and to 5000 for the MS/MS in the LTQ Velos, both with a maximum injection time of 200 ms.

The raw data were analyzed by MaxQuant software, version 1.2.2.5 for both protein identification and protein quantification. The Andromeda search engine was used to search the MS/MS spectra using the following parameters: trypsin/P with maximum 3 missed cleavage sites; peptide mass tolerance at first search was set at 20 ppm; MS/MS fragment mass tolerance at 0.50 Da, top 6 MS/MS peaks per 100 Da, and a minimum peptide length of 6 amino acids. Fixed modification for carbamidomethylation of cysteines and variable modifications for acetylation of protein N-termini, deamidation of asparagines and glutamines and oxidation of methionines were used. Identified peptides were searched against the available protein sequences from *S. flexneri* (SHIFL and SHIF8 protein databases) and *S. sonnei* (SHISS and SHISO databases) downloaded from UniProt (www.uniprot.org). One identified and quantified peptide was considered sufficient to call a protein present based on the fact that several predicted small proteins contained only 1 observable peptide. However most (2181 of 2306 proteins) were identified on the basis of multiple peptides (Table S1). False discovery rates were estimated based on the target-decoy approach (Balgley et al., 2007) using matches to reversed sequences in a concatenated database. The false discovery rate was set to 1% for proteins and peptides. Protein groups with a posterior error probability value over 0.01 or groups containing entries from the decoy database or contaminants were discarded. Since multiple databases were used, the software occasionally assigned homologs into different protein groups, e.g. where one detected peptide spanned a polymorphic region. To group genuine homologs and identify the protein group duplications, peptide IDs for each protein were matched with the peptide ID for all other proteins. The protein sequences of the members of the matching protein groups were then aligned to ensure that these were indeed genuine homologs and all members combined in a single protein group.

### 2.7 iBAQ individual protein quantification

The quantity of each protein was assessed as the Intensity-Based Absolute Quantification Index (iBAQ) (Schwanhäusser et al., 2011). The iBAQ of a protein/protein group is the sum of peak intensities of all peptides divided by the number of theoretically observable peptides. iBAQ values are approximately proportionate to the number of moles of protein present and thus iBAQ_*i*_/ΣiBAQ_*j*_ is the relative molar amount of protein *i*. In this paper we present results as the relative mass of protein present (m_*i*_), calculated for each protein *i* as: m_*i*_ = iBAQ_*i*_·M_*i*_/Σ(iBAQ_*j*_·M_*j*_), where M_*i*_ is the theoretical molecular weight of the protein *i* (Ishihama et al., 2005, Schwanhäusser et al., 2011).

### 2.8 Bioinformatics

The sequences of the identified proteins were analyzed by different bioinformatics tools. The prediction of the sub-cellular protein localization was carried out using LIPO (Berven et al., 2006) to look for inner and outer membrane lipoproteins. Proteins not identified as lipoproteins or scored as low probability lipoproteins were analyzed with PSORTb v3.0 (Yu et al., 2010). Finally, proteins still not assigned a definitive location were analyzed by SignalP 4.1 (Petersen et al., 2011) using a combination of artificial neural networks trained on proteins with signal peptides from Gram-negative bacteria. Proteins found to have a signal peptide were assumed to be periplasmic. 21 cases were manually checked, where peptides identified by mass spectrometry matched pairs of orthologs in the databases that had different predicted locations. For several of these, the different databases had assumed a different gene structure with different start codons. Where this occurred both members of the pair were assigned the same location based on the following: if one of the pair had a well-defined lipobox or signal peptide both members of the pair were assigned to a lipoprotein or periplasmic location, respectively. For proteins still unresolved, the pair was assigned the location of the member with the highest PSORTb prediction score.

For proteins that were identified only in *S. sonnei* 53G or only in *S. flexneri* 2a 2457T GMMA or Lysates the theoretical presence in the predicted proteome of the other strain was first assessed using protein BLAST (National library of Medicine, http://blast.st-va.ncbi.nlm.nih.gov/Blast.cgi). If no homologous protein was identified in the strain of interest, nucleotide BLAST of the corresponding gene was used. In silico translation (ExPASy Biofinformatics Resources Portal, http://web.expasy.org/translate/) was used to assess if a nucleotide BLAST hit is likely to be a functional protein coding gene (e.g. absence of frame shifts). For positive hits, a cut-off of 90% coverage and 95% identity of the protein sequence were used to call the protein theoretically present. The cut-off was based on the observed variation of proteins within selected protein groups.

The mass spectrometry proteomics data have been deposited to the ProteomeXchange Consortium (Vizcaino et al., 2014) via the PRIDE partner repository with the dataset identifier PXD002517.

## 3 Results

### 3.1 Composition of predicted outer membrane and periplasmic proteins in GMMA

**Fig. 1:**
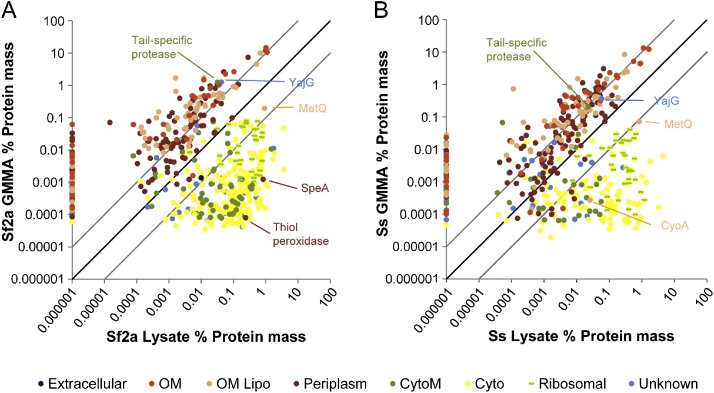
The relative abundance of proteins identified in GMMA compared to their abundance in whole cell lysates. (A) *S. flexneri* 2a and (B) *S. sonnei.* Proteins are plotted according to the percent of the estimated protein mass in the two samples and color coded according to the predicted location as follows: light orange, outer membrane lipoprotein; dark orange, integral outer membrane proteins; dark red, periplasmic protein; dark blue, extracellular; olive green, cytoplasmic membrane; yellow, cytoplasmic. Ribosomal proteins are distinguished from other cytoplasmic proteins by the green bar. Only proteins identified in GMMA are plotted. Proteins detected in GMMA but not in lysates were assigned 0.000001% mass and are plotted on the vertical axis. Proteins equally abundant in the GMMA and Lysate would be expected to lie on the dark diagonal line. The light diagonal lines above and below the identity line are guidance lines marking the theoretical positions of proteins enriched 10-fold in GMMA or 10-fold in the lysate, respectively. (For interpretation of the references to color in the figure legend, the reader is referred to the web version of this article.)

Analysis of solubilized whole cells and GMMA from the O antigen- and plasmid-negative *S. flexneri* 2a 2457T −p Δ*tolR* Δ*rfbG* (Sf2a Lysate and Sf2a GMMA respectively), and solubilized whole cell and GMMA from the O antigen and plasmid-negative *S. sonnei* 53G −p Δ*tolR* (Ss Lysate and Ss GMMA respectively) identified 2306 unique protein groups (in the following referred to as proteins) with an estimated abundance based on iBAQ quantification (Table S1, listed by the protein with the highest abundance in the protein group). Of these proteins, 2239 were found in at least one of the lysates, 659 of the proteins were identified in at least one of the GMMA samples. The protein abundance in GMMA closely followed the subcellular prediction made by a combination of LIPO (Berven et al., 2006), PSORTb (Yu et al., 2010), and SignalP (Petersen et al., 2011) with 95.7% and 98.7% of the protein mass found in the Sf2a and Ss GMMA, respectively, coming from predicted outer membrane (including outer membrane lipoproteins, OM lipo) or periplasmic proteins (Table 1). Of the 659 proteins found in GMMA, the 40 most abundant proteins account for 87.3% and 82.8% of the protein mass in Sf2a and Ss GMMA (Table 2). Compared to their abundance in the corresponding lysates, most of the outer membrane and periplasmic proteins are approximately 10-fold enriched in GMMA as a proportion of total protein (Fig. 1A and B). In accordance with the enrichment in GMMA, they comprise only 8.3% of the protein mass in Sf2a Lysates and 11.4% in Ss Lysates, respectively.

The non-outer membrane and non-periplasmic protein category was dominated by similar proteins for both Sf2a and Ss GMMA. Small quantities of ribosomal proteins collectively comprise more than half of the mass of the cytoplasmic proteins in GMMA (Table 1 and Table S2). In addition, Tail-specific protease (E3Y2N8_SHIFL) predicted to be located at the cytoplasmic membrane and YajG, a putative polymerase/proteinase (Q3Z4W9_SHISS) with no definitive subcellular localization, are the major contributors to the non-outer membrane/periplasmic components (Table 1).

There were a few proteins that show an anomalous distribution, expected to be GMMA components from the predicted locations but were preferentially found in the lysate (Fig. 1 and Table S1). Predicted outer membrane lipoproteins MetQ (d-methionine-binding lipoprotein MetQ, E7K3R2_SHISO) and CyoA (Ubiquinol oxidase, subunit II, E7K828_SHISO), were more abundant in lysates than in GMMA (MetQ: 0.91% and 0.82% in Sf2a and Ss Lysates vs 0.19% and 0.07% in Sf2a and Ss GMMA, respectively; CyoA: 0.0255% in Ss lysate vs 0.0003% in Ss GMMA). In contrast, outer membrane predicted thiol peroxidase (E3Y2F2_SHIFL) and SpeA (Biosynthetic arginine decarboxylase, Q3YXT3_SHISS) were only detected in Sf2a GMMA although they were prominent in both lysates. In addition, ten predicted periplasmic proteins were found in both the Sf2a and Ss Lysates, but not in either of the GMMA samples (Table S1).

Proteins of specific interest in GMMA were iron-regulated outer membrane proteins as they elicited protection in animal models against *E. coli* (Fox et al., 2009), *Salmonella* (Kaneshige et al., 2009), and *Bordetella pertussis* (Alvarez Hayes et al., 2013). Approximately 2.4% of the protein mass were comprised by iron-uptake proteins in both Ss and Sf2a GMMA. Aerobactin siderophore receptor IutA (E7T7F9_SHIFL), Ferrichrome-receptor FepA (E3YA60_SHIFL) and Ferrichrome-iron receptor FhuA (E7K3K6_SHISO) were present in both strains. CirA and iron (III) dicitrate transport protein FecA (E7K2N5_SHISO) were additionally detected in Ss GMMA (Table S1).

In order to measure the enrichment of proteins in the GMMA compared to the whole cell lysate, we did non-weighted linear regressions of the log-transformed percentages of each predicted outer membrane and periplasmic protein in GMMA versus lysate for proteins with a mass abundance of greater than 0.01%. This gave slopes of 0.83 ± 0.12 and 0.85 ± 0.09 for the Sf2a and Ss preparations, respectively. At the mid-points of the regression, there was a predicted 13.3-fold enrichment (95% confidence interval 9.1–19.5) in Sf2a GMMA and 8.3-fold (6.5–10.7) in Ss GMMA. Neither slope was significantly different from 1, consistent with a similar enrichment of all (across the abundance range) predicted periplasm and outer membrane proteins in GMMA (Fig. 1). There was no statistically significant difference in the enrichment of membrane proteins compared to periplasmic proteins in either the Sf2a or Ss preparations (*p* = 0.94 and 0.85 by *t* test, for Sf2a and Ss, respectively).

### 3.2 Comparative analysis of Sf2a and Ss GMMA and lysates

**Fig. 2:**
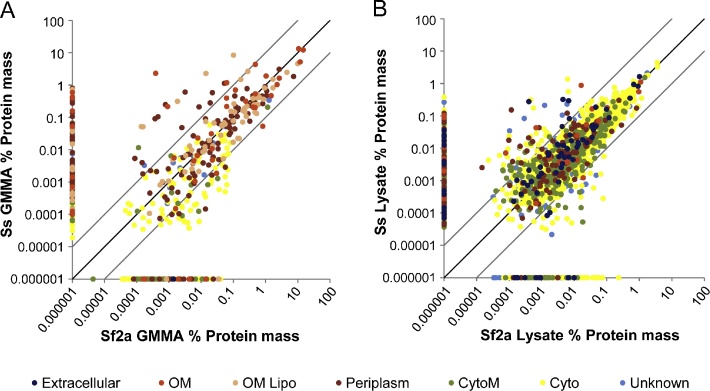
The relative abundance of proteins identified in Ss GMMA compared to Sf2a GMMA (A) and in Ss Lysate compared to Sf2a Lysates (B). The color codes are as described in the legend for Fig. 1, except ribosomal proteins are not explicitly marked but are part of the cytoplasmic proteins. Proteins detected in GMMA or Lysates from only 1 strain were assigned 0.000001% mass in the other strain and plotted on the axes. The light diagonal lines above and below the identity line are guidance lines marking the theoretical positions of proteins enriched 10-fold in Ss or 10-fold in Sf2a samples, respectively. (For interpretation of the references to color in the figure legend, the reader is referred to the web version of this article.)

By comparing the 4 samples, we assessed the consistency of the samples. Of the 659 proteins identified in at least one of the GMMA samples, 290 (44%) were common to both and these accounted for 99.6% and 94.9%, of the protein mass in Sf2a GMMA and Ss GMMA, respectively. Similarly of the 2239 proteins identified in at least one of the lysates, 1696 were common (76%) and these accounted for 99.0% and 96.4% of the total protein mass in the Sf2a lysate and Ss lysate, respectively. Significantly, the abundance of the common proteins was highly correlated in each of the pairs of samples. Direct correlation plots (Fig. 2) show that the majority of proteins lie close to the identity line and the Pearson correlation coefficients were 0.80 and 0.91 for GMMA and lysates, respectively (*p* < 0.0001 for both).

There were a small number of proteins that were relatively more abundant in the Ss GMMA than in the Sf2a GMMA, including some proteins of moderate abundance that were only seen in the Ss GMMA (Table 3). Approximately half of these proteins were confirmed to be absent in Sf2a by genome analysis (Table 3). In contrast, there were only 4 moderately abundant proteins with predicted outer membrane or periplasmic localization in Sf2a GMMA that were not found in Ss GMMA of which by genome analysis only 1 protein would be expected to be present in Sf2a (Table 4).

For proteins detected both in Sf2a and Ss we assessed the reproducibility of protein abundance in the Sf2a and Ss Lysate based on the log ratio of iBAQ. Considering the common 1696 proteins, the distribution of the log ratios closely followed a normal distribution with a best fit (minimized sum of errors squared) mean of 0.0054 with SD of 0.42 (Fig. S1A). Thus, for the 1696 proteins the average correlation of the % mass was 1.01 (antilog of 0.0054). The impact of the magnitude of the iBAQ on the mean and SD of the log ratios was tested by dividing the protein population into 10 groups of increasing iBAQ. No variation in the log ratio or SD was seen in groups with geometric mean iBAQ ranging from 10,000 to 10,000,000 (Fig. S1B). This is consistent with the individual abundance plots in Fig. 2B and previous studies using iBAQ (Schwanhäusser et al., 2011). There was a small deviation with approximately 4.5% of Ss proteins with an average of 10× greater abundance that the corresponding Sf2a proteins (Fig. S1A and Table S1), similar to the findings in GMMA (Table 3).

We investigated the technical reproducibility by measuring the log ratio of iBAQ and corresponding SD for pairs of ribosomal proteins in the two lysate samples. We assume that all ribosomes have stoichiometric amounts of each protein and also that there is negligible amounts of ribosomal protein not in ribosomes. Thus variation in the ratio of the iBAQ for the 53 pairs of ribosomal proteins in the two lysates is a measure of the technical reproducibility of the methods (Table S2). The ratio of molar abundance of ribosomal proteins in the 2 samples had on average, a ratio of 1.11. Thus the ratio of ribosomal proteins in the two samples was similar to the ratios of all proteins (1.11 vs 1.01), but the standard deviation of the log ratio was lower for the ribosomal proteins compared to all proteins (0.22 vs 0.42).

The accuracy of using iBAQ to estimate the molar concentration of proteins was assessed by comparing the iBAQ values of the individual ribosomal proteins within each lysate (Table S2), again assuming a negligible amount of free ribosomal proteins, as ribosomal proteins should be present in the same molar quantity regardless of the size variation. Similar to other studies (Arike et al., 2012), there was appreciable variation in the molar estimates with the 95% confidence interval calculated from the log transformed iBAQ covering a 10.7- and 8.5-fold range for the Sf2a and Ss lysates, respectively.

Since we were able to show that most proteins are present in approximately equal relative quantities in the Ss and Sf2a Lysates, we used the frequency at which the orthologs were detected in both the Ss and Sf2a Lysates to estimate detection limit of this assay. First, for each protein that was detected only in either the Ss or the Sf2a Lysates, we checked if the gene was present in both genomes and was likely to be functional (i.e. absence of frame shift mutations). This resulted in 2017 proteins where at least one was detected and both Ss and Sf2a potentially had functional genes. We ranked the proteins in order of increasing individual iBAQ or average iBAQ (where both where present), and then divided the set into 40 groups of increasing iBAQ. We counted the percentage of cases where proteins were detected in both samples (conditional on detecting at least one) and plotted this against the mean iBAQ of the group (Fig. S2). We assumed that the probability (*p*) of detecting a protein in a single sample was a simple hyperbolic function of iBAQ (*p* = iBAQ/(iBAQ + iBAQ_0.5_), where iBAQ_0.5_ is the iBAQ with a 50% probability of detection. We then did an unweighted fit of the percentage of cases where proteins were detected in both samples (% two proteins detected = 100*p*^2^/(2(1 − *p*)*p* + *p*^2^) to iBAQ, minimizing the sum of error squared. This gave a good fit (*r*^2^ = 0.754) with an iBAQ_0.5_ of 6134.

## 4 Discussion

In previous studies that identified proteins in outer membrane blebs (SOMV, NOMV and GMMA), proteins with predicted outer membrane or periplasmic localization accounted for half or fewer of all identified proteins (Berlanda Scorza et al., 2008, Berlanda Scorza et al., 2012, Choi et al., 2011, Lappann et al., 2013, Mullaney et al., 2009, Park et al., 2011, Pierson et al., 2011, Roier et al., 2015, van de Waterbeemd et al., 2013, Zybailov et al., 2006). In the study presented here, we also found less than half of all proteins to have predicted outer membrane or periplasmic localization, with 265 of 659 proteins identified in either Sf2a or Ss GMMA. Analyzing the protein content quantitatively gives a different view. As measured by iBAQ, nearly all of the protein content in the GMMA is predicted to be derived from outer membrane or periplasmic localized proteins: in this study 95.7% and 98.7% of the protein content in Sf2a and Ss GMMA, respectively.

The most abundant outer membrane proteins in the GMMA were identified as OmpA, OmpC, and OmpX consistent with previous observations with Ss GMMA (Berlanda Scorza et al., 2012) and with the preparation of GMMA from cultures in the early exponential phase (Zhu et al., 2007). Surprisingly, Entericidin B and not Lpp (Braun, 1975), the most abundant lipoprotein in *E. coli*, was the most abundant lipoprotein in Ss GMMA (8.4%) while it comprised only 0.1% mass in Sf2a GMMA. This difference could be related to the difference in the media composition.

A total of 60 samples (15 gel slices of each of the 4 preparations, Fig. S3) were used in this study and the large number of samples made technical or biological replicates problematic. Therefore, the estimates of the reliability of the study relies on the consistency between the Sf2a and Ss GMMA samples, between the Sf2a and Ss Lysate samples and between the corresponding GMMA and Lysate samples. Several measures of the reproducibility and precision were included in the analysis.

Estimates of the relative mass of the proteins depends on the assumption that iBAQ is a useful measure of the relative molar quantities of the proteins present. Analysis of the iBAQ values of ribosomal proteins within a sample provides an estimate of the accuracy in this study: the calculated molar ratios varied with a 95% confidence interval of approximately 9.6-fold (Table S2) in line with other studies (Arike et al., 2012, Maier et al., 2011, Ishihama et al., 2008). Although the uncertainty of individual protein abundances is relatively large, when averaged over large numbers of proteins the error in the estimate, e.g. of cytoplasmic contamination in GMMA, will be small. Comparison of the ribosomal data between the Sf2a and Ss samples showed that the reproducibility between samples is good. The variance of the log ratios of the ribosomal proteins was 0.049 (i.e. 95% confidence interval of 2.7-fold) but the variance of the 1696 common proteins in the lysate was 0.17. This suggests that approximately a quarter of the variation between the samples from Sf2a and Ss results from variation within the method, e.g. sample preparation or the mass spectrometry, whereas three quarters of the variance is a true difference between the Sf2a and Ss Lysates, reflecting differential abundance of specific proteins.

There are two groups of proteins where the reproducibility between samples was much less than expected: 4.5% of proteins were approximately 10× overexpressed in Ss compared to Sf2a. In addition, 11 of the 191 protein present in the Ss GMMA at an abundance of >0.01% were not detected in Sf2a despite the corresponding gene being present in Sf2a and 10 of the 191 proteins were >100 times more abundant than in Sf2a GMMA. In contrast, only 1 protein present in Sf2a GMMA at >0.01% of total protein was not detected in Ss GMMA though the gene is present (Table 3, Table 4). The iBAQ score in the Ss or Sf2a GMMA for the proteins absent in GMMA from the other strain ranged from 326,930 to 10,197,000. If the detection limit for the GMMA samples was similar to that assessed on the Lysate, the corresponding probability of detecting these proteins ranged from 0.965 to 0.999. Therefore, it is unlikely that these were present at a similar abundance and missed by chance. Of the proteins that were highly enriched in Ss GMMA compared to Sf2a GMMA, 3 are related to maltose metabolism: maltose-binding periplasmic protein (E7JZV4_SHISO), a homologue of MalE (Chapon, 1982), maltose operon periplasmic protein (E7JZV7_SHISO), a homologue of MalM (Gilson et al., 1986), and maltoporin LamB (LAMB_SHISS). The corresponding genes are part of the *malT* operon and subject to catabolite repression (Chapon, 1982) and thus the addition of glucose to the growth medium for Sf2a but not for Ss is a possible reason for the low or absent expression of these three genes. For the other proteins, the reason for the relative abundance in Ss is unclear.

Since the lysates are solubilized whole cells, all the proteins found in GMMA should also be in the lysates, albeit at lower relative abundance. Comparison of the Sf2a GMMA with the Sf2a Lysate and Ss GMMA with the Ss Lysate (Fig. 1) confirmed this assumption and demonstrated that most of the proteins detected in the GMMA samples were also present in the lysates at approximately the same abundance ranks. Moreover, comparison of Sf2a Lysate with Ss Lysate showed that most of the proteins were conserved in the samples from the two strains and that on average were present at the same abundance (Fig. 2B). Similarly, the common proteins in Sf2a GMMA with Ss GMMA accounting for 99.6% and 94.9% of the protein mass, respectively, were present in close to the same mass rank order.

In both the Sf2a and Ss GMMA, approximately 20% of the protein was predicted to be periplasmic. This was twice the value for outer membrane blebs from *P. aeruginosa* (10% (Choi et al., 2011)) and three times that for *N. meningitidis* (6.2% (Lappann et al., 2013)) suggesting that the *Pseudomonas* NOMV and *Neisseria* SOMV had a larger surface to volume ratio than the *Shigella* GMMA in this study. A surprising observation in the current study was the approximately 10-fold enrichment of both periplasmic and outer membrane proteins in GMMA compared to total cell proteins (Fig. 1A and B). This suggests that the GMMA co-incidentally have a similar surface to volume ratio as the surface to periplasmic volume ratio of the bacteria. Electron microscopy of hyperblebbing bacteria with the *tolR* mutation showed that the separation between inner and outer membrane is substantially greater than in the wild-type bacteria (Meloni E, unpublished data). Thus, the genetic enhancement bleb formation may be linked to the enrichment of periplasmic proteins in Sf2a and Ss GMMA (approximately 20% in both) compared to spontaneously released vesicles from *P. aeruginosa* (10% (Choi et al., 2011)) and *N. meningitidis* (6.2% (Lappann et al., 2013)).

The “purity” of the GMMA with respect to outer membrane and periplasmic proteins is likely to be even higher than the initial analysis suggested: Two proteins, Tail-specific protease and YajG, highly enriched in GMMA but not predicted to be outer membrane or periplasmic proteins (Fig. 1) appear to be misclassified using the sequences from the *Shigella* database. Tail-specific protease is known to be a soluble periplasmic protease in *E. coli* (Keiler and Sauer, 1996) and the YajG homologue in *E. coli* is a lipoprotein with a classic LIPO box (Boudet et al., 2007). Taking this into account, 98.1% and 99.3% of Sf2a and Ss GMMA, respectively, would be periplasmic or outer membrane localized proteins. Much of the remaining protein content is ribosomal proteins. The GMMA used in this study were purified by ultracentrifugation under conditions that contaminating ribosomes, if present in the medium as a result of low-level bacterial lysis, are likely to have been co-sedimented with the GMMA. It will be interesting to see if GMMA purified using filtration, for use in vaccines are also contaminated with ribosomes.

These results are different to the data from naturally shed outer membrane vesicles from *N. meningitidis* where 85.8% of the protein mass in the SOMV was predicted to be periplasmic (6.2%) or outer membrane (79.6%) (Lappann et al., 2013). For *P. aeruginosa* NOMV the prediction was even lower with approximately 47% of the protein in the vesicles predicted to be periplasmic or outer membrane (Choi et al., 2011). Importantly, the cytoplasmic and inner membrane predicted proteins that would be considered impurities in an outer membrane bleb-based vaccine accounted for 13.9% in *Neisseria* SOMV (Lappann et al., 2013) or 38% in *Pseudomonas* NOVM (Choi et al., 2011) in contrast to 4.4% in Sf2a GMMA and only 1.3% in Ss GMMA (or only 1.9% and 0.7%, respectively, assuming misclassification of Tail-specific protease and YajG).

It is unlikely that these differences relate to differences in the surface polysaccharide content or LPS/LOS structure: The *Pseudomonas* was presumably encapsulated (not specified in the paper), but the *Neisseria* had the capsule deleted and neither *Shigella* lines were encapsulated (Caboni et al., 2015). By deleting the O antigen, the LPS of the *Shigella* lines had a similar size and structure to the LOS present in the *Neisseria*. The *Pseudomonas* and the *Shigella* had a wild type Lipid A component of their LPS, the Neisseria contained the *lpxL* mutation that results in penta-acylated lipid A. It is possible that the differences observed in these studies relates to the use of the *tolR* mutation in *Shigella* that result in a very high levels of blebbing. By substantially increasing the production of GMMA, the level of impurities in the resulting GMMA preparation is likely to be substantially reduced. This is a critical point for vaccine production: it suggests that cytoplasmic proteins are not intrinsically bound to GMMA and therefore their presence needs to be carefully controlled.

In conclusion, using quantitative proteomics, we show that we can produce GMMA from *S. sonnei* and *S. flexneri* 2a that contain the expected outer membrane and periplasmic proteins and have the low levels of cytoplasmic protein contamination. This makes their composition and purity suitable for vaccines.

## Figures and Tables

**Table 1:** Predicted location of proteins found in Sf2a and Ss GMMA and Lysates.

Predicted location	GMMA				Lysates			
	% mass		Number of proteins		% mass		Number of proteins	
	Sf2a	Ss	Sf2a	Ss	Sf2a	Ss	Sf2a	Ss
Outer membrane								
Outer membrane integral proteins (OM)	47.85	48.12	33	39	3.37	5.04	22	33
Outer membrane lipoprotein (OM lipo)	28.00	32.34	66	75	2.72	4.05	52	59
Periplasmic	19.86	18.25	105	133	2.2	2.28	90	127
**Total predicted outer membrane/periplasmic**	**95.70**	**98.71**	**204**	**247**	**8.29**	**11.37**	**164**	**219**
Cytoplasmic								
Ribosomal	0.86	0.28	43	35	15.03	15.08	53	53
Other cytoplasmic	0.63	0.20	177	93	65.08	61.30	1137	1198
Cytoplasmic membrane								
Tail-specific protease	1.15	0.21	1	1	0.03	0.02	1	1
Other cytoplasmic membrane	0.24	0.13	53	29	7.00	6.44	291	346
Extracellular	0.00	–	1	0	0.02	0.01	5	4
Unknown								
YajG	1.28	0.34	1	1	0.04	0.05	1	1
Other unknown	0.13	0.13	35	28	4.51	5.72	216	245
**Total predicted non-OM/periplasmic**	**4.30**	**1.29**	**311**	**187**	**91.71**	**88.63**	**1704**	**1848**

**Table 2:** 40 most abundant proteins found in GMMA (by average % mass in Sf2a and Ss GMMA).

Gene name	Location	Protein name	Uniprot name	Average % mass in GMMA	% mass in Sf2a GMMA	% mass in Ss GMMA
SF2457T_0298	OM	Outer membrane protein C	E3XX60_SHIFL	13.411	14.619	12.203
SF2457T_0799	OM	Outer membrane protein A	E3XYK2_SHIFL	11.943	10.615	13.271
SS53G_3941	OM	Outer membrane protein X	E9UMG3_SHISO	8.506	11.829	5.183
SS53G_0415	OM lipo	Repeated sequence found in lipoLPP family prot.	E7JU63_SHISO	7.261	9.955	4.566
SF2457T_4977	PP	Protease Do (HtrA)	E3YA49_SHIFL	4.604	7.366	1.842
SS53G_2965	OM lipo	Entericidin B	E7K193_SHISO	4.257	0.101	8.413
SS53G_0370	OM lipo	Outer membrane lipoprotein pcp	E7JU20_SHISO	3.528	4.570	2.485
tolB	PP	Protein tolB	TOLB_SHISS	2.859	2.637	3.080
SS53G_0760	OM	Outer membrane porin protein LC	E7JV54_SHISO	2.419	0.479	4.358
SF2457T_3748	OM	Outer membrane protein	E3Y6Q5_SHIFL	2.145	2.654	1.636
yaeT	OM	Outer membrane protein assembly factor yaeT	YAET_SHISS	1.646	2.258	1.035
lpoA	OM lipo	Penicillin-binding protein activator LpoA	LPOA_SHIF8	1.595	1.679	1.510
SF2457T_0832	OM	Outer membrane protein F	E3XYN5_SHIFL	1.471	1.036	1.905
SGF_01778	OM lipo	Glycoprotein-polysaccharide metabolism	E7TB57_SHIFL	1.465	0.280	2.650
SS53G_1454	OM lipo	Osmotically inducible lipoprotein B	E7JX36_SHISO	1.256	1.692	0.820
lamB	OM	Maltoporin	LAMB_SHISS	1.156	0	2.312
slp	OM lipo	OM protein induced after carbon starvation	Q3YWI0_SHISS	1.151	0.014	2.288
SGF_00427	OM	Aerobactin siderophore receptor IutA	E7T7F9_SHIFL	0.977	1.486	0.468
SF2457T_4357	PP	Peptidyl-prolyl cis-trans isomerase	E3Y8E7_SHIFL	0.932	0.968	0.896
pal	OM lipo	Peptidoglycan-associated lipoprotein	E7K8X6_SHISO	0.873	0.930	0.816
SS53G_1748	PP	Putative uncharacterized protein	E7JXX8_SHISO	0.869	1.080	0.658
SS53G_0544	OM lipo	SmpA/OmlA family protein	E7JUJ2_SHISO	0.829	0.170	1.489
yajG	Unknown	Putative polymerase/proteinase	Q3Z4W9_SHISS	0.808	1.276	0.340
SF2457T_2094	PP	Periplasmic oligopeptide-binding protein	E3Y260_SHIFL	0.769	1.065	0.473
SF2457T_2279	CytoM	Tail-specific protease	E3Y2N8_SHIFL	0.679	1.152	0.206
surA	PP	Chaperone surA	SURA_SHISS	0.623	0.787	0.458
SF2457T_0435	OM	Long-chain fatty acid outer membrane transporter	E3XXF4_SHIFL	0.622	0.062	1.181
SS53G_1935	OM lipo	SmpA/OmlA family protein	E7JYG3_SHISO	0.611	0.858	0.364
degQ	PP	Serine endoprotease	Q3YX14_SHISS	0.592	0.053	1.132
SS53G_3947	PP	Putative uncharacterized protein	E9UMG9_SHISO	0.540	0.816	0.265
SF2457T_3200	OM lipo	Pectinesterase B	E3Y553_SHIFL	0.532	0.651	0.414
SF2457T_3582	OM lipo	Peptidase family M48 family protein	E3Y5Y2_SHIFL	0.530	0.555	0.504
livJ	PP	High-affinity amino acid transport system	Q3YW68_SHISS	0.521	0.264	0.777
yeaF	OM	Putative uncharacterized protein yeaF	Q3Z2C1_SHISS	0.507	0.479	0.535
SS53G_4569	OM lipo	Putative phospholipid-binding domain protein	E7K576_SHISO	0.468	0.535	0.401
SF2457T_4988	OM	Ferrichrome-iron receptor	E3YA60_SHIFL	0.435	0.816	0.053
SF2457T_4129	PP	PP dipeptide transport protein	E3Y7S0_SHIFL	0.434	0.401	0.467
ybjP	OM lipo	Putative enzyme	Q0T8Q3_SHIF8	0.414	0.429	0.400
SF2457T_3629	OM	Serine protease eatA	E3Y628_SHIFL	0.410	0.115	0.705
yfgL	OM-Lipo	Outer membrane assembly lipoprotein YfgL	E7JUN3_SHISO	0.409	0.571	0.246

**Table 3:** Proteins preferentially found in Ss GMMA.

Gene name	Location	Protein name	Uniprot name	% mass in Ss GMMA	Ratio	Theoretically present in Sf2a*
**Predicted outer membrane and periplasmic localized proteins with at least 0.01% mass in Ss GMMA not found in Sf2a GMMA**						
SS53G_2461	PP	Maltose-binding periplasmic protein	E7JZV4_SHISO	0.799	−**	+
SS53G_0290	OM lipo	TonB-dependent Receptor Plug domain protein	E7JTU0_SHISO	0.732	−	−
SS53G_3463	OM	Iron(III) dicitrate transport protein fecA	E7K2N5_SHISO	0.589	−	−
cirA	OM	OM receptor for iron-regulated colicin I receptor	Q3Z051_SHISS	0.430	−	−
exc	OM lipo	Entry exclusion protein 2	A4SH43_SHISS	0.423	−	−
SS53G_3761	OM	Ferrichrome-iron receptor	E7K3K6_SHISO	0.396	−	−
SS53G_2464	PP	Maltose operon periplasmic protein	E7JZV7_SHISO	0.337	−	+
ycdO	PP	UPF0409 protein ycdO	YCDO_SHISS	0.276	−	+
SS53G_0127	OM	Putative tonB-dependent receptor yncD	E7JTC8_SHISO	0.159	−	−
hdeA	PP	Putative uncharacterized protein hdeA	Q3YWI4_SHISS	0.140	−	+
mdoG	PP	Glucans biosynthesis protein G	OPGG_SHISS	0.106	−	+
SS53G_2146	PP	Putative uncharacterized protein	E7JZ10_SHISO	0.064	−	−
SS53G_0128	PP	PQQ enzyme repeat family protein	E7JTC9_SHISO	0.058	−	−
SS53G_5268	OM	TonB-dependent Receptor Plug domain protein	E7K7A5_SHISO	0.053	−	+
SS53G_2796	OM lipo	Inner membrane lipoprotein yiaD	E7K0S5_SHISO	0.051	−	+
SSON_1625	PP	Putative sulfatase	Q3Z1P2_SHISS	0.047	−	−
SS53G_0368	OM lipo	Putative uncharacterized protein	E7JU18_SHISO	0.036	−	−
SS53G_4157	PP	Putative uncharacterized protein	E7K461_SHISO	0.032	−	+
yehZ	PP	Putative transport system permease protein	Q3Z073_SHISS	0.026	−	+
SS53G_4973	PP	Acid-resistance protein	E7K6I7_SHISO	0.025	−	+
SS53G_0106	PP	Bacterial extracellular solute-binding family protein	E7JTA7_SHISO	0.024	−	−
SSON_1637	PP	Putative hemin-binding lipoprotein	Q3Z1N0_SHISS	0.022	−	−
tauA	PP	Taurine transport system periplasmic protein	Q3Z543_SHISS	0.019	−	−
SS53G_3203	OM lipo	Putative uncharacterized protein	E7K1X5_SHISO	0.015	−	+

Predicted outer membrane and periplasmic localized proteins highly enriched in Ss GMMA compared to Sf2a GMMA***						
lamB	OM	Maltoporin	LAMB_SHISS	2.312	6017	
slp	OM lipo	Outer membrane protein induced after carbon starvation	Q3YWI0_SHISS	2.288	163	
glpQ	PP	Glycerophosphodiester phosphodiesterase, periplasmic	Q3YZW7_SHISS	0.609	193	
SS53G_4155	OM lipo	Putative polysaccharide export protein gfcE	E7K459_SHISO	0.313	170	
SS53G_3546	PP	Putative phospholipid-binding domain protein	E7K2W6_SHISO	0.249	321	
SS53G_4156	OM	Putative uncharacterized protein	E7K460_SHISO	0.216	239	
SS53G_5756	PP	Glutamate/aspartate periplasmic-binding protein	E7K8P1_SHISO	0.105	230	
ycdB	PP	Peroxidase ycdB (Deferrochelatase/peroxidase EfeB)	YCDB_SHISS	0.091	246	
ymcC	OM lipo	Putative regulator	Q3Z3D5_SHISS	0.052	376	
blc	OM lipo	Outer membrane lipoprotein	Q3YUI9_SHISS	0.051	329	

* Present if protein sequence with ≥90% coverage and ≥95% identity was identified in Sf2a proteome or translated genome.

** Protein not detected in Sf2a GMMA.

*** Proteins >0.01% total protein mass and with at least 100× higher % mass in Ss GMMA than Sf2a GMMA.

**Table 4:** Proteins preferentially found in Sf2a GMMA.

Gene name	Location	Protein name	Uniprot name	% mass in Sf2a GMMA	Theoretically present in Ss*
**Predicted outer membrane and periplasmic localized proteins with at least 0.01% mass in Sf2a GMMA not found in Ss GMMA**					
SF2457T_1008	OM lipo	Uncharacterized lipoprotein ydcL	E3XZ64_SHIFL	0.035	−
SF2457T_0155	OM lipo	Lipoprotein	E3XWR9_SHIFL	0.035	−
sitA	PP	Iron transport protein	Q3Z1C5_SHISS	0.024	+
SF2457T_3658	OM	Antigen 43	E3Y656_SHIFL	0.014	−

* Present if protein sequence with ≥90% coverage and ≥95% identity was identified in Ss proteome or translated genome.

## Appendix A Supplementary data

Table S1 The table lists the 2306 proteins identified in the 4 samples tested: *S. flexneri* 2a GMMA (Sf2a GMMA), *S. flexneri* 2a lysate (Sf2a Lysate), *S. sonnei* GMMA (Ss GMMA) and *S. sonnei* lysate (Ss Lysate), by gene name, UniProt name, predicted location, protein name, molecular weight, sequence length, peptides detected and with the calculated relative abundance expressed as a % moles and as % mass.
