# Supplementary material for: Quantitative proteomic analysis of Shigella flexneri and Shigella sonnei Generalized Modules for Membrane Antigens (GMMA) reveals highly pure preparations
Source: Int J Med Microbiol. 2016 Feb;306(2):99–108. doi: 10.1016/j.ijmm.2015.12.003 (PMC4820968; doi:10.1016/j.ijmm.2015.12.003)
Supplement: Supplementary file 2 [file mmc2.docx]

**Table S2: Ribosomal proteins found in Sf2a and Ss GMMA and Lysates**

|  | **GMMA** | | | | **Lysate** | | | |
| --- | --- | --- | --- | --- | --- | --- | --- | --- |
|  | **% moles** | | **% mass** | | **% moles** | | **% mass** | |
| **Protein Name** | **Sf2a** | **Ss** | **Sf2a** | **Ss** | **Sf2a** | **Ss** | **Sf2a** | **Ss** |
| 30S ribosomal protein S1 | 0.0004 | 0 | 0.0012 | 0 | 0.406 | 0.3742 | 0.8475 | 0.8076 |
| 30S ribosomal protein S2 | 0.0172 | 0.0007 | 0.0205 | 0.0009 | 0.5565 | 0.4259 | 0.5056 | 0.4 |
| 30S ribosomal protein S3 | 0.0222 | 0.0039 | 0.0257 | 0.0053 | 0.843 | 0.648 | 0.7434 | 0.5907 |
| 30S ribosomal protein S4 | 0.0206 | 0.001 | 0.0215 | 0.0012 | 0.6084 | 0.7583 | 0.4846 | 0.6244 |
| 30S ribosomal protein S5 | 0.0008 | 0.0002 | 0.0006 | 0.0001 | 0.5453 | 0.6327 | 0.3258 | 0.3908 |
| 30S ribosomal protein S6 | 0 | 0 | 0 | 0 | 0.0057 | 0.0097 | 0.003 | 0.0052 |
| 30S ribosomal protein S7 | 0.0019 | 0.0003 | 0.0017 | 0.0003 | 0.9629 | 1.6925 | 0.6547 | 1.1896 |
| 30S ribosomal protein S8 | 0 | 0 | 0 | 0 | 0.1731 | 0.1447 | 0.083 | 0.0717 |
| 30S ribosomal protein S9 | 0.0129 | 0.0247 | 0.0085 | 0.019 | 0.2567 | 0.3033 | 0.1295 | 0.1581 |
| 30S ribosomal protein S10 | 0.0045 | 0.004 | 0.0024 | 0.0024 | 1.6536 | 1.0111 | 0.6586 | 0.4163 |
| 30S ribosomal protein S11 | 0.0018 | 0.003 | 0.0011 | 0.0021 | 0.8956 | 0.8109 | 0.4209 | 0.3939 |
| 30S ribosomal protein S12 | 0.0002 | 0 | 0.0001 | 0 | 0.0403 | 0.0366 | 0.0188 | 0.0176 |
| 30S ribosomal protein S13 | 0.0606 | 0.0442 | 0.0353 | 0.03 | 1.7235 | 1.4277 | 0.7663 | 0.6561 |
| 30S ribosomal protein S14 | 0.0002 | 0 | 0.0001 | 0 | 0.0474 | 0.2586 | 0.0186 | 0.1051 |
| 30S ribosomal protein S15 | 0.0104 | 0.0003 | 0.0047 | 0.0001 | 0.3336 | 0.2336 | 0.1163 | 0.0842 |
| 30S ribosomal protein S16 | 0 | 0 | 0 | 0 | 1.4192 | 1.115 | 0.5513 | 0.4478 |
| 30S ribosomal protein S17 | 0.1573 | 0.0048 | 0.0678 | 0.0024 | 0.7206 | 0.3905 | 0.2374 | 0.1329 |
| 30S ribosomal protein S18 | 0.1995 | 0.089 | 0.0796 | 0.0414 | 1.788 | 1.3517 | 0.5453 | 0.4262 |
| 30S ribosomal protein S19 | 0 | 0.0013 | 0 | 0.0007 | 0.2027 | 0.0713 | 0.0718 | 0.0261 |
| 30S ribosomal protein S20 | 0.0087 | 0.0222 | 0.0037 | 0.0111 | 0.1232 | 0.1738 | 0.0404 | 0.0589 |
| 30S ribosomal protein S21 | 0.1167 | 0.051 | 0.0441 | 0.0225 | 1.4726 | 1.8854 | 0.4249 | 0.5623 |
| 50S ribosomal protein L1 | 0.0045 | 0.001 | 0.005 | 0.0012 | 0.1975 | 0.4451 | 0.1658 | 0.3862 |
| 50S ribosomal protein L2 | 0.0028 | 0.0002 | 0.0037 | 0.0003 | 0.1509 | 0.1801 | 0.1529 | 0.1887 |
| 50S ribosomal protein L3 | 0.0018 | 0 | 0.0018 | 0 | 0.6785 | 0.4066 | 0.5123 | 0.3173 |
| 50S ribosomal protein L4 | 0.0006 | 0.0001 | 0.0006 | 0.0001 | 0.7022 | 0.8068 | 0.5262 | 0.6249 |
| 50S ribosomal protein L5 | 0.0053 | 0.0005 | 0.0047 | 0.0005 | 1.11 | 1.2945 | 0.7649 | 0.922 |
| 50S ribosomal protein L6 | 0.0017 | 0 | 0.0014 | 0 | 0.6467 | 0.5611 | 0.4149 | 0.3721 |
| 50S ribosomal protein L7/L12 | 0.0022 | 0 | 0.0012 | 0 | 0.3813 | 0.124 | 0.1591 | 0.0535 |
| 50S ribosomal protein L9 | 0.0009 | 0 | 0.0006 | 0 | 0.5812 | 0.6309 | 0.3111 | 0.3491 |
| 50S ribosomal protein L10 | 0.0067 | 0.0024 | 0.0053 | 0.0022 | 0.6097 | 0.7885 | 0.3665 | 0.49 |
| 50S ribosomal protein L11 | 0.0008 | 0 | 0.0005 | 0 | 0.1756 | 0.1488 | 0.0887 | 0.0776 |
| 50S ribosomal protein L13 | 0.0727 | 0.0079 | 0.0518 | 0.0066 | 0.3596 | 0.5379 | 0.1957 | 0.3026 |
| 50S ribosomal protein L14 | 0.0036 | 0 | 0.0022 | 0 | 0.7797 | 0.5577 | 0.3583 | 0.2649 |
| 50S ribosomal protein L15 | 0.1075 | 0.0101 | 0.0714 | 0.0079 | 0.4949 | 0.4944 | 0.2514 | 0.2596 |
| 50S ribosomal protein L16 | 0.0819 | 0.0246 | 0.0556 | 0.0194 | 0.3451 | 0.5118 | 0.179 | 0.2744 |
| 50S ribosomal protein L17 | 0.1295 | 0.0221 | 0.0826 | 0.0164 | 0.5092 | 0.4627 | 0.2483 | 0.2332 |
| 50S ribosomal protein L18 | 0.0246 | 0.0005 | 0.0139 | 0.0004 | 0.3238 | 0.1927 | 0.1403 | 0.0863 |
| 50S ribosomal protein L19 | 0.0024 | 0.0013 | 0.0014 | 0.0009 | 0.961 | 0.6228 | 0.4284 | 0.287 |
| 50S ribosomal protein L20 | 0.1069 | 0.0165 | 0.0642 | 0.0115 | 0.1509 | 0.2218 | 0.0693 | 0.1052 |
| 50S ribosomal protein L21 | 0.0907 | 0.0057 | 0.0466 | 0.0034 | 0.0783 | 0.2823 | 0.0307 | 0.1145 |
| 50S ribosomal protein L22 | 0.0844 | 0.0297 | 0.0459 | 0.0188 | 1.251 | 0.9267 | 0.5198 | 0.398 |
| 50S ribosomal protein L23 | 0.0138 | 0.0113 | 0.0069 | 0.0065 | 0.0979 | 0.0607 | 0.0372 | 0.0238 |
| 50S ribosomal protein L24 | 0 | 0 | 0 | 0 | 0.3605 | 0.64 | 0.1384 | 0.2541 |
| 50S ribosomal protein L25 | 0 | 0 | 0 | 0 | 0.1307 | 0.1112 | 0.06 | 0.0528 |
| 50S ribosomal protein L27 | 0 | 0 | 0 | 0 | 0.6204 | 0.3367 | 0.1921 | 0.1078 |
| 50S ribosomal protein L28 | 0.1399 | 0.0643 | 0.056 | 0.03 | 0.5914 | 0.5109 | 0.1808 | 0.1614 |
| 50S ribosomal protein L29* | 0.0002 | 0.003 | 0.0001 | 0.0011 | 0.0342 | 0.029 | 0.0084 | 0.0074 |
| 50S ribosomal protein L30 | 0.0051 | 0.0193 | 0.0015 | 0.0065 | 0.7015 | 0.5659 | 0.1557 | 0.1299 |
| 50S ribosomal protein L31 | 0 | 0 | 0 | 0 | 0.0977 | 0.1975 | 0.0261 | 0.0545 |
| 50S ribosomal protein L32 | 0 | 0 | 0 | 0 | 0.9149 | 1.0263 | 0.2002 | 0.2321 |
| 50S ribosomal protein L33 | 0 | 0 | 0 | 0 | 0.2415 | 0.2765 | 0.0522 | 0.0618 |
| 50S ribosomal protein L34 | 0.0366 | 0.0221 | 0.0087 | 0.0062 | 0.0239 | 0.0535 | 0.0044 | 0.0101 |
| 50S ribosomal protein L35 | 0.0808 | 0.0069 | 0.0283 | 0.0028 | 1.6734 | 1.1502 | 0.4481 | 0.3184 |

*Different proteins (uniprot names) for L29 in Sf2a and Ss: Sf2a E3Y8G8_SHIFL, Ss RL29_SHISS

| **A**  **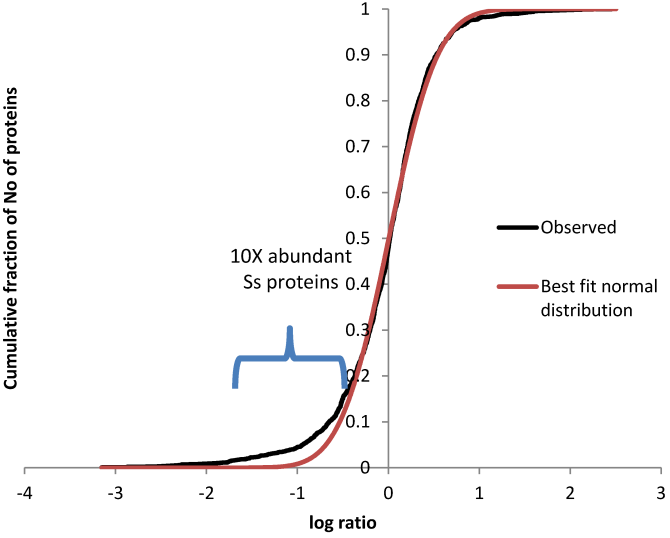** | **B**  **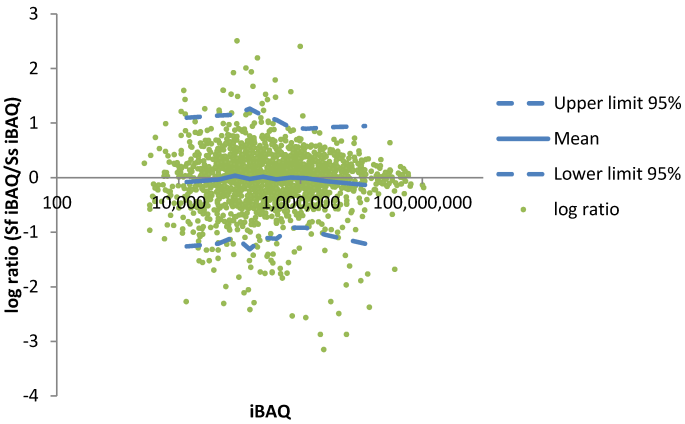** |
| --- | --- |

**Figure S1: Reproducibility of protein abundance in the Sf2a and Ss Lysate based on the log ratio of iBAQ**

**A.** Log ratios (Sf2a iBAQ/Ss iBAQ) of the proteins detected in both Lysates were plotted as cumulative fraction of the number of proteins and the best fit normal distribution was calculated (minimized sum of errors squared). A small deviation from the best fit was observed around the log ratio of -1 reflecting about 4.5 % of proteins that are approximately 10-fold abundant in Ss versus Sf2a. **B.** Log ratios (Sf2a iBAQ/Ss iBAQ) of the proteins detected in both Lysates were plotted against the average iBAQ. The set was divided into 10 groups of increasing iBAQ and mean log ratios and upper and lower 95% confidence intervals were calculated for each group and indicated in the graph.


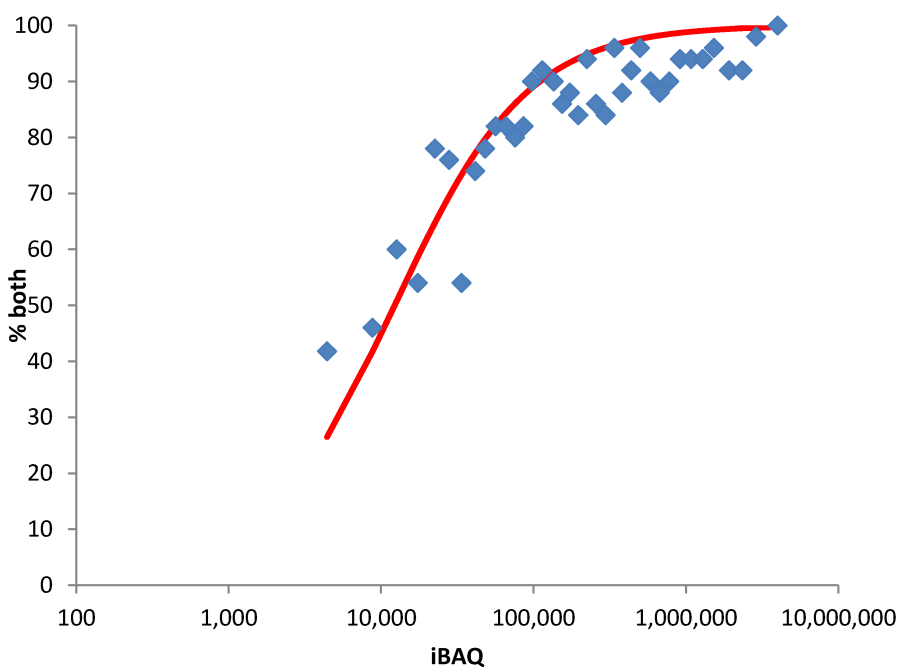


Figure S2. Probability of detecting a protein in both lysates, Sf2a and Ss, dependent on the iBAQ

2017 proteins identified in either or both of the Sf2a and Ss Lysates, and for which a potentially functional gene was identified from the genomic analysis of both genomes were ranked in order of increasing individual iBAQ or average iBAQ (where both where present), and then divided into 40 groups of increasing iBAQ. The percentage of cases in where proteins were detected in both was plotted against the mean iBAQ of the group. The observed points fit a binomial distribution, assuming that the probability of detection of a single protein in a single sample is a hyperbolic function,
*p*= iBAQ/(iBAQ + iBAC*_0.5_*) where iBAQ*_0.5_* is the iBAQ with a 50% probability of detection in a single sample. The percent of detecting both in two samples as a proportion of detecting at least one is then 100*p*^2^/(2(1-*p*)*p*+*p*^2^). The best fit is for an iBAQ*_0.5_* of 6134, r^2^ =0.754.

**Supplementary Figure 3**


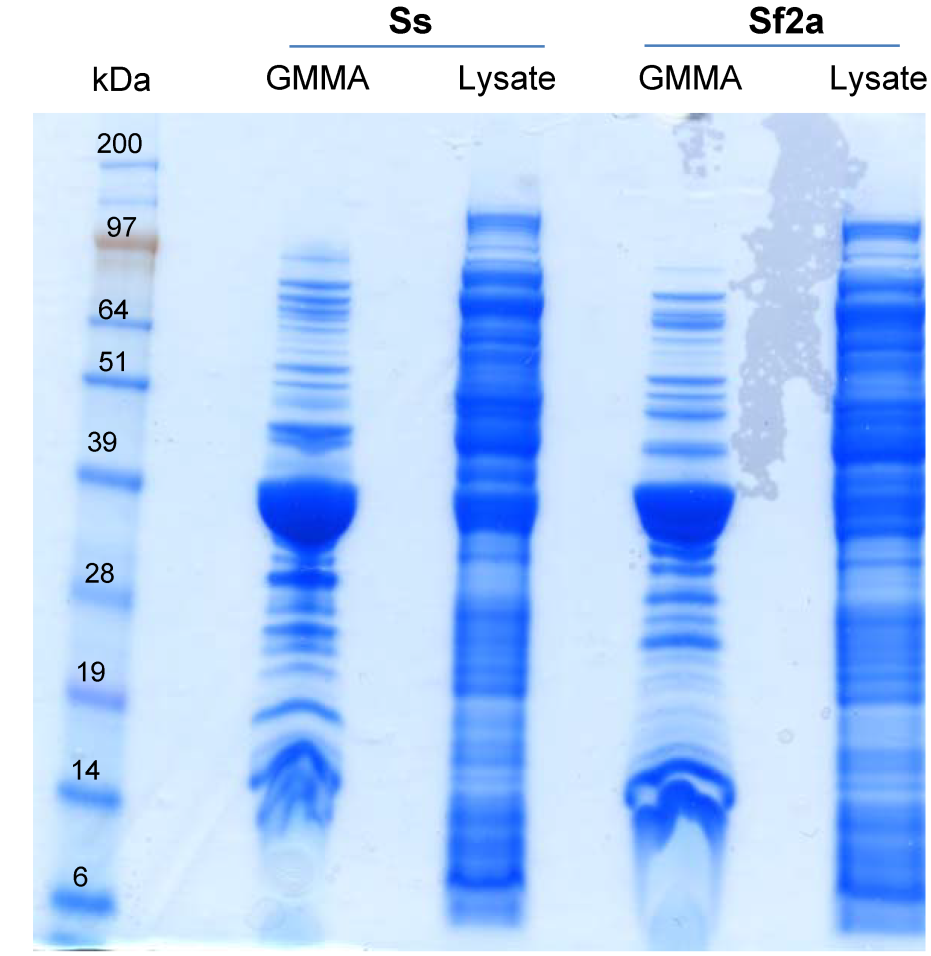


Figure S3: SDS gel of the GMMA and lysate preparations.
